# New water-soluble colorimetric pH and metal ione sensor based on graphene quantum dot modified with alizarine red S

**DOI:** 10.1038/s41598-020-70821-5

**Published:** 2020-08-25

**Authors:** Hassan Ahmadi, Sajjad Keshipour, Fatemeh Ahour

**Affiliations:** Department of Nanochemistry, Nanotechnology Research Center, Urmia University, Urmia, Iran

**Keywords:** Synthesis of graphene, Environmental, health and safety issues, Quantum dots, Synthesis and processing

## Abstract

A new colorimetric sensor was designed for the screening pH changes in solutions, as well as, detection of some cations. The sensor preparation includes the chemical binding of alizarine red S (ARS) as a sensor of pH and cation to graphene quantum dots (GQD). Loading ARS on GQD led to the formation of water soluble sensor which finally responded to the colorimetric detection of some cations in water. Solubility and stability of the sensor in water indicate that the sensor is an ideal system for the biological and environmental applications. To demonstrate the applicability of the new sensor, the colorimetric responds of sensor were examined for some cations including Fe^3+^, Co^2+^, Ca^2+^, As^3+^, Cd^2+^, Hg^2+^, Pb^2+^, Sn^2+^, Al^3+^, and Cr^3+^. The colorimetric detections of all the ions were performable individually in a solution. In addition, GQD-ARS as a colorimetric sensor detected Co^2+^ at pH < 0.6 with limit of quantification 0.08 mM and Fe^3+^ at 0.6 < pH < 4.0 with limit of quantification 0.03 mM in the mixture of cations.

## Introduction

Colorimetric chemosensing technique is a promising detection approach of organic and inorganic species with naked eye. Colorimetric chemosensors are test kits for onsite detection with some considerable characteristics such as fast detection, simplicity, reversibility, high selectivity, and excellent sensitivity. The pH responsive functional materials have broad applications including wearable^1,2^ and textilebased chemical sensors^3^, smart food packaging^4,5^, food freshness monitoring^6^, and non-invasive bioprocess monitoring^7^. Optical sensors based on fluorescence materials have attracted great attention because of advantages over conventional electrochemical approaches especially due to ease of detection by the naked eye, and high sensitivity^8–10^. While many optical sensors have been reported, stimuli-responsive polymer-based optical sensors^11–14^ including polymeric micelles^15–19^ and nanoparticle/polymer hybrids^18,20–24^ offer benefits over the classical optical sensors having fast response time and reversible changes in the conformations of the polymers. The disadvantages of these systems are the disability to sense responses over a wide range of pH and temperature, and insolublity or poor dispersion stability in aqueous media^25^. Optical sensor based on graphene oxide (GO) exhibits distinctive ratiometric color responses. GO has been identified as a promising sensing platform with high signal-to-noise ratio for stimuli responsive optical sensor because of its excellent potential as a highly sensitive Förster resonance energy transfer (FRET) acceptor^26–28^. Recently, a colorimetric GO-based pH sensor was introduced that responds to a wide range of pH changes^29^. The system is a hybrid of responsive polymer and quantum dot (QD) integrated on a single GO sheet. Moreover, modified GOQDs–poly(vinyl alcohol) hybrid hydrogels as solid sensing platform, colorimetrically detected Fe^2+^, Co^2+^ and Cu^2+^ in aqueous media^30^. Detection of cationic species is an important domain of supramolecular chemistry due to their relevance in medicinal, environmental, and biological fields^31,32^. Nile red dispersed on partially oxidized graphene oxide by ultrasonication selectively detected and identified Fe(III) specie with color change from purple to dark brown^33^.

For designing a colorimetric sensor, two or more florescent chromophores are required with ability to emmit different colors with strong luminescence. Semiconducting QDs have been used extensively for the preparation of colorimetric sensors because of their high quantum yields, size-dependent photoluminescence (PL) emissions, and multiple emissions with a single light source^34–38^. A number of reports with single PL intensity-based QD-anchored GO based optical sensors have been developed with sensing elements, such as chitosan, the molecular beacon, and poly(aniline)^39,40^. To the best of our knowledge, GQDs-based colorimetric sensors was limited to a few reports^41,42^. Herein we synthesized a GQD-based optical sensor with distinctive ratiometric color responses. The colorimetric GQDs-based sensors were synthesized from the chemical modification of GQDs with alizarin red S (ARS) as a chormophor. GQDs exhibit excellent solubility in aqueous media which can be useful for the ion detection in aqueous systems. While all of the previous mentioned reports are hybrid systems based on mixing various chemicals, the present report is the chemical binding ARS to GQD to achieve a new sensor.

## Results and discussion

**Figure 1:**
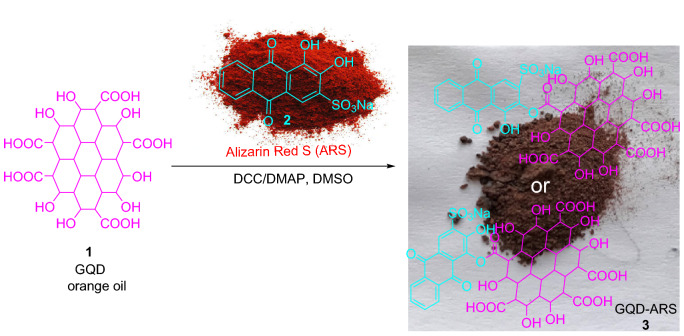
Modification of GQD with ARS.

Figure 1 shows our key strategy for the synthesis of GQD-ARS as colorimetric ion sensor. The pH-responsive compound, ARS, was bonded to GQDs via activation with *N*,*N'*-dicyclohexylcarbodiimide (DCC) and *N*,*N'*-dimethylaminopyridine (DMAP)^43^. ARS has active hydroxyl groups for the reaction with GQD through the esterification reaction which two products conceivable (**3**). The reaction of orange oil GQD with red color ARS gives dark brown powder as the GQD-ARS.

**Figure 2:**
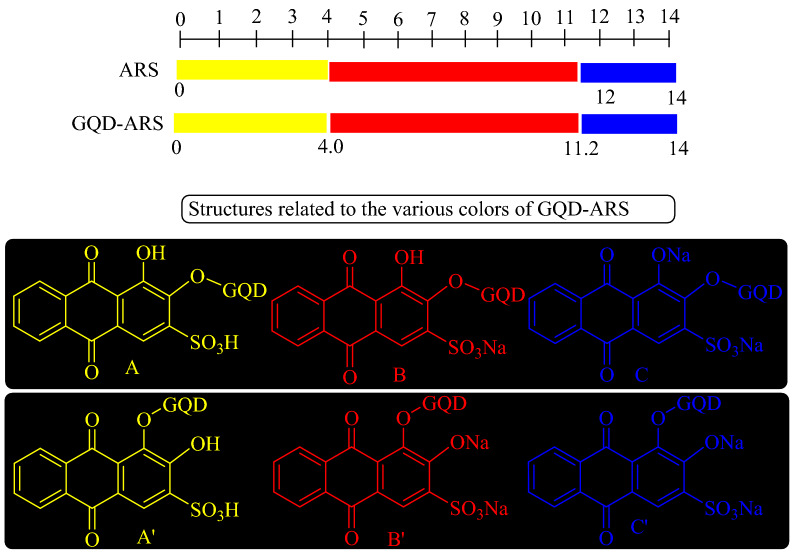
Color and structure changes for GQD-ARS in various pHs.

The new GQD-based sensor responded over a wide range of pH values. Three distinctive colors were observed for GQD-ARS in various screened pHs (Fig. 2), while the GQD has orange color in all pH values. GQD-ARS showed yellow color at pH < 4.0 similar to ARS (A and Aʹ in Fig. 2). With increasing pH from 4.0, the color of sensor was changed to red which attributed to the proton elemination from the sulfonic moiety (B and Bʹ in Fig. 2) which obviously demonstrated the sulfonyl group did not participate in the reaction with GQD. At pH above 11.2, proton elimination was occurred from the hydroxyl group of sensor leading to the purple color of the solution (C and Cʹ in Fig. 2).

**Figure 3:**
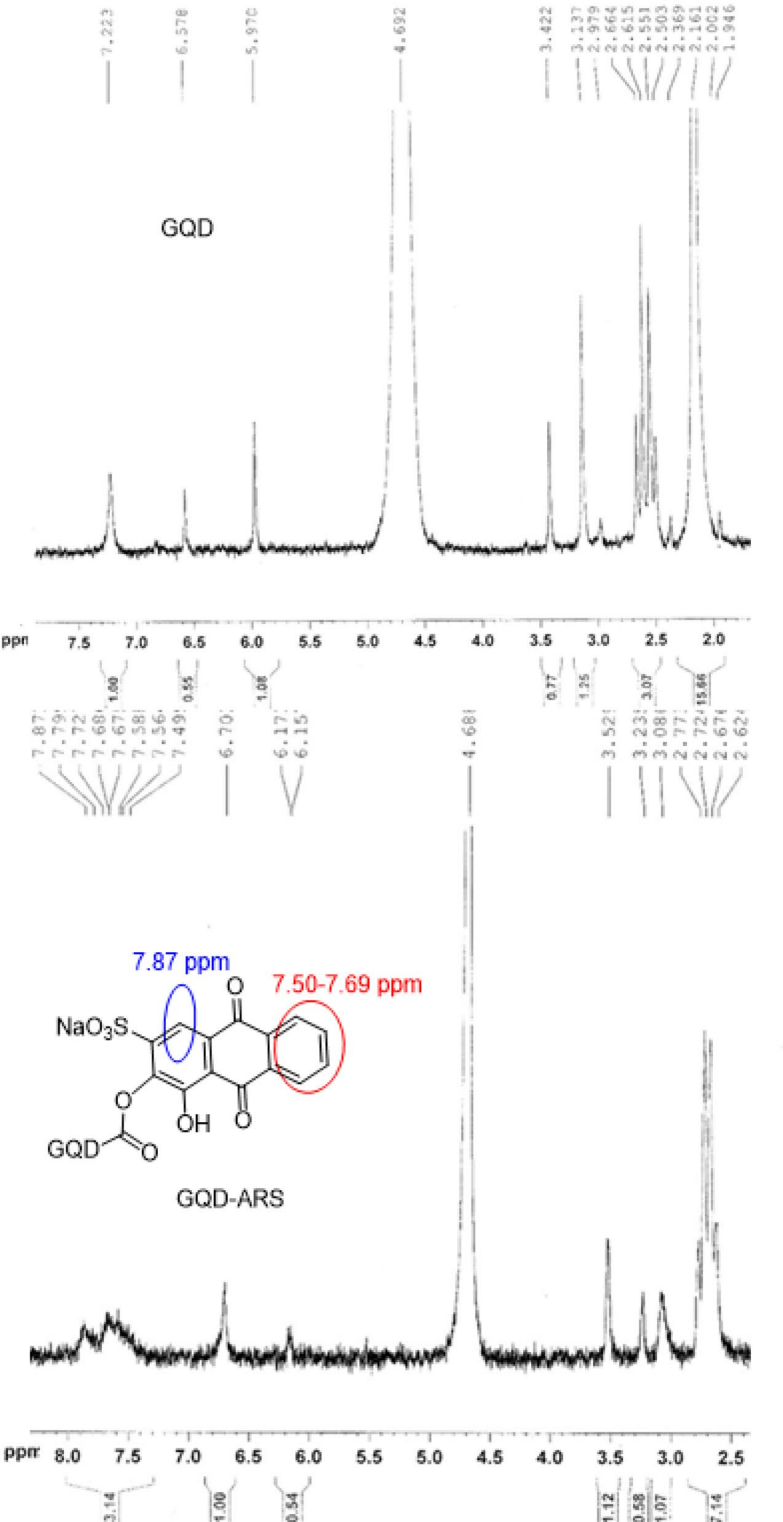
^1^H NMR of GQD and GQD-ARS.

The chemical modification of GQDs with ARS was approved with ^1^H NMR (Fig. 3). ^1^H NMR of ARS-GQD showed Ar–H which attributed to the ARS loaded on GQD. Appearance of the new signals at 7.50–7.69 and 7.87 ppms in ^1^H NMR spectrum of GQDs-ARS compared to GQD spectrum obviously confirmed the modification process (Fig. 3).

**Figure 4:**
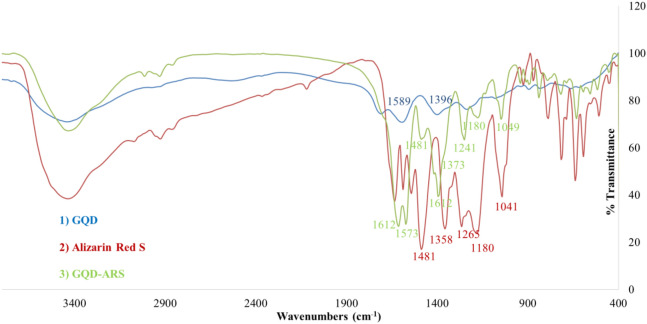
FT-IR for compounds **1**–**3**.

FT-IR spectrum of GQD-ARS indicated absorption peaks attributed to the ARS, and GQD structures. For example, the absorption bands at 1573, and 1611 cm^−1^ for GQD-ARS were related to C=O functionality of GQD. Although the peaks attributed to GQD on GQD-ARS were appeared in high intensity, the absorption bands of ARS on GQD-ARS revealed weakly. The peaks at 1,049, 1,180, 1,241, 1,373, and 1,481 cm^−1^ in the FT-IR spectrum of GQD-ARS are related to the ARS moiety (Fig. 4).

**Figure 5:**
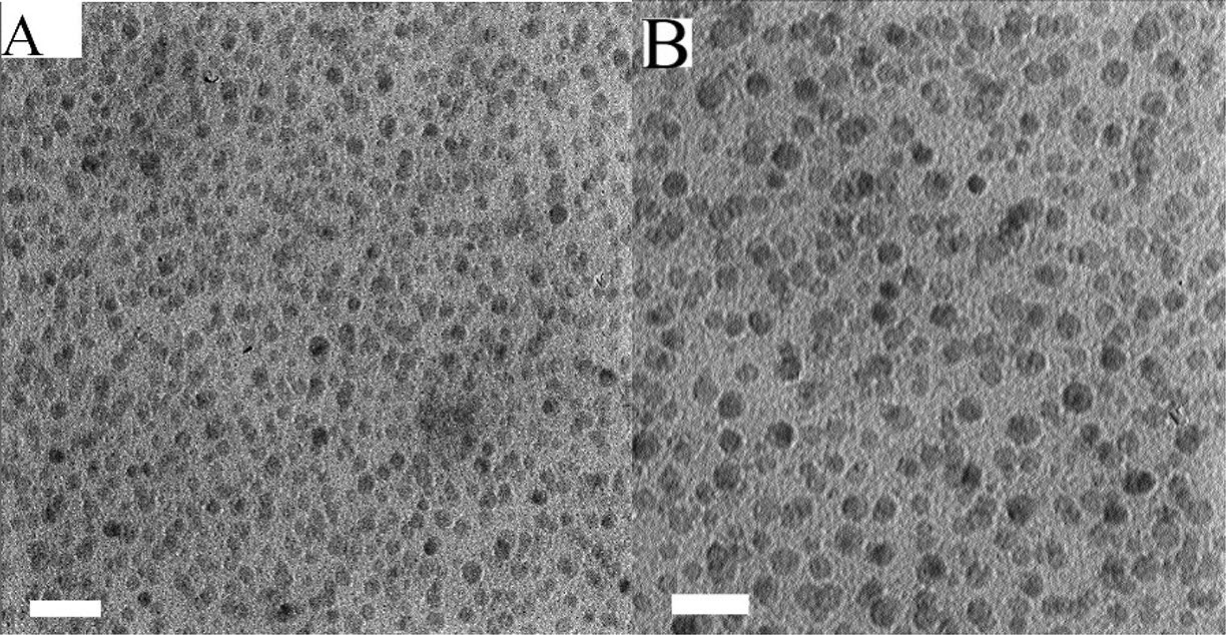
TEM micrographs of GQD-ARS (The scale bar is 100 nm; **A**) and GQD (The scale bar is 50 nm; **B**).

TEM micrgraph was prepared for GQD-ARS (Fig. 5). TEM image showed formation of very homogeneous nanoparticles of GQD with maximum size distribution between 15 and 20 nm.

**Figure 6:**
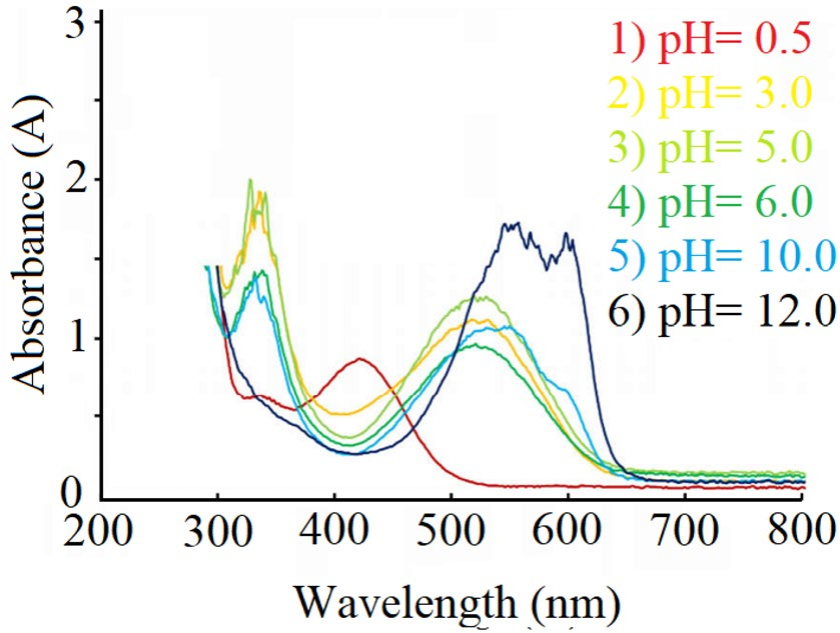
UV–Vis spectra of GQD-ARS in different pHs.

While ARS has low solubility in water, GQD-ARS is soluble in water which attributed to the water solubility of GQD. Water solubility is important characteristic for a sensor, since the water soluble sensors can be used in the bio and environmental systems. Figure 6 shows the UV–Vis spectra of GQD-ARS in aqueous media (0.05 g/mL) at different pH conditions. GQD demonstrated an absorption peak at 351 nm which its intensity was increased at high pHs because of the transformation of carboxylic acids belong to GQD to carboxylates. Carboxylate groups increase the electron density on GQD due to the negative charge which leads to the easy excitation phenomenon and finally augmentation of the peak intensity. The UV–Vis spectrum of yellow solution GQD-ARS showed two absorption peaks about at 335, and 420 nm at pH below 0.6. With increasing pH, the absorption peak at 420 nm was faded and the intensity of peak at 335 nm was gradually increased. Two peaks at about 335, and 520 nm were observed at 0.6 < pH < 4.0 and the color of the solution was still remained yellow. The solution had red color at 4.0 < pH < 11.2, but the UV–Vis spectra were different below and above pH = 10. While two absorption peaks at about 335 and 520 nm were observed below pH = 10, at higher pH a new absorption peak was added at about 600 nm, the peak at 520 nm moved to about 540 nm, and the peak at about 335 nm weakened. Above pH = 11.2 the color was changed completely to purple with two peaks at about 550 nm and 600 nm. Therefore, the λ_max_ of GQD-ARS was strongly related to the pH. The proposed structures of GQD-ARS in pH < 4.0, 4.0 < pH < 11.2, and pH > 11.2 are shown in Fig. 2. These structures are proposed based on ARS structures in various pHs.

The specific and strong interactions of ARS with metal ions as well as specific optical properties of the GQDs directed us to use GQD-ARS as a new optical sensor for metal ions detections. GQD-ARS showed pH-related colors for some of the screened metal ions, including Fe^3+^, Co^2+^, Ca^2+^, As^3+^, Cd^2+^, Hg^2+^, Pb^2+^, Sn^2+^, Al^3+^, and Cr^3+^ (Table 1). For this purpose, solutions with different pH values including 0.5, 3, 7, and 12 were prepared which GQD-ARS had indicated different UV–Vis spectra in these pHs. The color changes were recorded after addition of the synthesized indicator to the 0.2 mM solution of metal ions at different pH values (Table 1) regarding that the obsorved color changes were because of the complexes formations between metal ions and GQD-ARS. The results evinced that GQD-ARS is a pH-related colorimetric sensor for metal ions. The color of GQD-ARS solution in pH = 0.5 was changed from yellow to green with addition of Co^2+^, indicated Co^2+^ ions successfully formed complex with GQD-ARS. GQD-ARS showed low color changes for the Co^2+^ solution in pH = 3 compared to standard solution containing GQD-ARS without metal ion. Another color change to purple was observed at pH = 7 although the resulted color was similar to the color change observed for other cations. Therefore, GQD-ARS is a colorimetric sensor for the detection of Co^2+^ specie in solutions with pH < 0.6. The limit of quantification (LOQ) for Co^2+^ by naked eyes was obtained 0.08 mM in pH < 0.6.

**Table 1:**
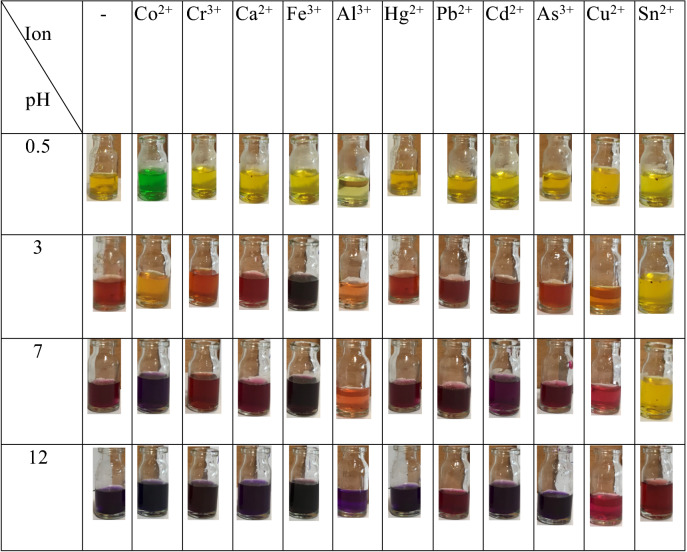
Color changes of GQD-ARS in the absence and presence of various metal ions.

Colorimetric determination of Fe^3+^ can be performed by GQD-ARS with the color change from yellow to purple at 0.6 < pH < 4.0. The addition of Fe^3+^ to GQD-ARS solution converted the color of mixture from red to dark purple. The limit of quantification (LOQ) for Fe^3+^ by naked eyes was obtained 0.03 mM.

GQD-ARS ability was surveyed in the selective detection of Co^2+^ and Fe^3+^ metal ions. For this purpose, two mixtures were prepared in pH = 0.5, and 3 containing all of the investigated cations and GQD-ARS was added to these mixtures. The color of the mixtures changed to green and purple, respectively. As mentioned above, Co^2+^ gave green color to the solution containing GQD-ARS at pH < 0.6 and also the color of ions mixture was remained green at this pH with the same absorption spectra. The color of solution in the absence of Co^2+^ was yellow revealing GQD-ARS ability to detect Co^2+^ selectively in the mixture of ions investigated here at pH < 0.6. GQD-ARS also can be used for the selective detection of Fe^3+^ in the presence of other mentioned metal ions at pH 3 due to the color change from yellow to purple. GQD-ARS cannot be used for the selective detection and determination of other ions since most of them gave same color to GQD-ARS containing solution. So, GQD-ARS is colorimetric responsive indicator for Co^2+^ and Fe^3+^ in a mixture of ions.

**Figure 7:**
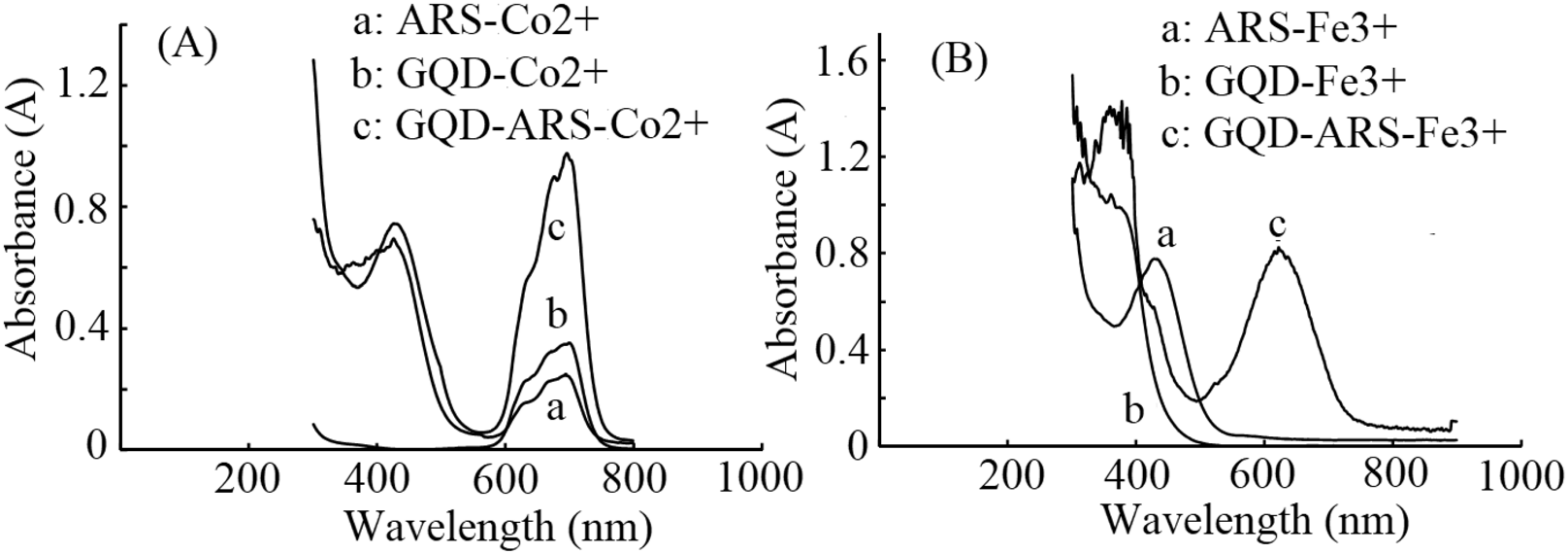
UV–Vis spectra of (**A**) GQD, ARS, and GQD-ARS-Co^2+^ (**B**) GQD, ARS, and GQD-ARS-Fe^3+^

Under the optimum conditions (pH < 0.6 for Co^2+^ and pH = 3 for Fe^3+^), the colorimetric assay was processed using GQD-ARS to detect a series of Co^2+^ and Fe^3+^ solutions with concentrations greater than 1 µM. The detectios according a obvious color change from yellow to green for Co^2+^ and yellow to purple for Fe^3+^ ions were also observable by UV–Vis absorption spectroscopy. The obtained spectra for GQD, ARS and GQD-ARS in the presence of Co^2+^ and Fe^3+^ have been shown in Fig. 7.

**Figure 8:**
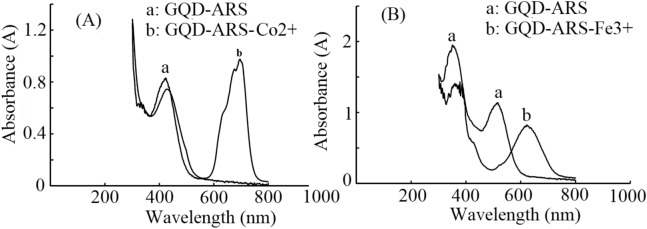
UV–Vis spectra of (**A**) GQD-ARS and GQD-ARS-Co^2+^ (**B**) GQD-ARS and GQD-ARS-Fe^3+^

Figure 7 confirmed the color changes of the solution and proved the ability of GQD-ARS for the detection of Co^2+^ and Fe^3+^ in the optimized pH condition. The distinctions in the absorption spectra of GQD-ARS with and without Fe^3+^ and Co^2+^ in the optimized pHs are great values. The position of maximum band, λ_max_, undergoes a shift which is particularly noticeable (Fig. 8) could be used for sensitive and selective detections of Fe^3+^ and Co^2+^ in the certain pH.

**Figure. 9:**
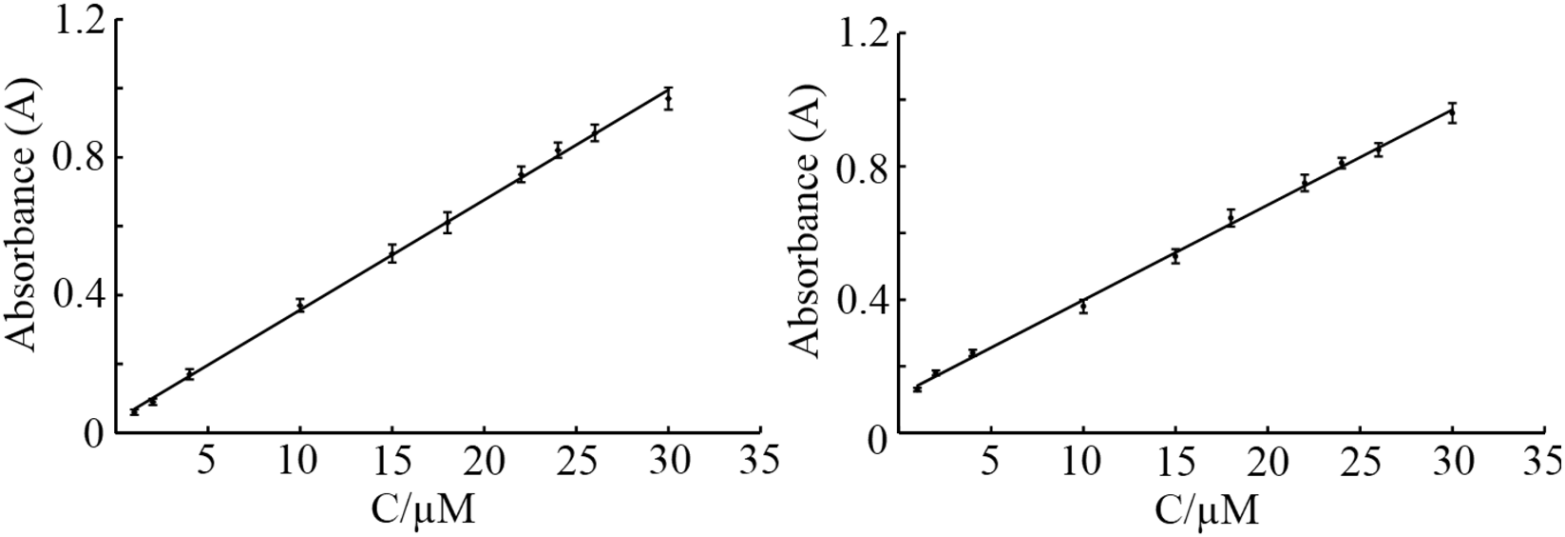
Variation of absorbance signal versus concentration (calibration curve) for (**A**) Co^2+^, and (**B**) Fe^3+^.

Under optimum condition the absorption of the solution was measured by addition different concentration of Co^2+^ and Fe^3+^ ions and results showed that (Fig. 9) there is a linear regression between absorbance and concentration in the range from 1 to 30 µM for these cations.

The obtained correlation coefficient were 0.999 and 0.998 for Co^2+^ and Fe^3+^ ions, respectively. The calculated detection limit based on signal to noise ratio (S/N = 3) was calculated to be 0.31 µM and 0.35 µM for Co^2+^ and Fe^3+^ cations. Detailed studies for the colorimetric determination of these cations using GQD-ARS are presently in progress in our laboratory and results will be reported soon.

## Conclusion

A novel water-soluble sensor was synthesized through modification of GQD with ARS which characterized with common methods. While ARS is insoluble compound in water, GQD-ARS is completely soluble and can be interesting sensor in biosystems. The sensor demonstrated color change in four pH ranges including pH < 0.6, 0.6 < pH < 4.0, 4.0 < pH < 11.2, and pH > 11.2. GQD-ARS also showed great performance in the pH-related colorimetric detection of some metal ions. The sensor particularly determines Co^2+^ and Fe^3+^ in the mixture of ions.

## Experimental

### Materials and methods

All reagents were purchased from Merck or Aldrich and used without further purification. Fourier transform infrared spectroscopy (FTIR) was used to characterize different functional groups of the composite using a Jasco 6,300 FTIR instrument in the range of 600–4,000 cm^−1^. Transition electron microscopy (TEM) micrographs were obtained with Philips CM100 BioTWIN transmission electron microscope and TEM Philips EM 208S. ^1^H NMR Spectra were recorded on a Bruker DRX-300 Avance spectrometer 300.13 MHz; chemical shifts (δ scale) are reported in parts per million (ppm). UV–Visible spectrophotometer of Biowave II, Biochrom WPA Ltd., UK was used for preparation of absorption spectra.

### Preparation of GQD-ARS

For the preparation of GQD, citric acid (2.0 g) was heated at 180 °C during 15 min to give orange oil (1.1 g) which purified with addition of 20 ml acetone and 20 ml NaOH solution (0.1 N) to the mixture and separation of oil part of the mixture^44^.

GQD-ARS was synthesized from stirring GQD (2.0 g), ARS (0.1 g), DCC (0.4 g), and DMAP (0.05 g) in DMSO (10 ml) at 90 °C. After 24 h, the mixture was cooled to room temperature, acetone (10 ml) was added to the reaction vessel, and the precipitate was filtered off. Finally, washing the obtained solids with acetone (2 × 5 ml) and drying at 60 °C gives GQD-ARS as a dark brown (1.8 g).

### General procedure for the detection of ions

To a pH adjusted solution of M^x+^ (5 ml, 0.2 mM) with HCl or NaOH, GQD-ARS (0.05 g) was added and the color of solution was changed immediately.
